# Low Dosage of Histone H4 Leads to Growth Defects and Morphological Changes in *Candida albicans*

**DOI:** 10.1371/journal.pone.0010629

**Published:** 2010-05-13

**Authors:** Lucia F. Zacchi, Anna M. Selmecki, Judith Berman, Dana A. Davis

**Affiliations:** 1 Department of Microbiology, University of Minnesota, Minneapolis, Minnesota, United States of America; 2 Department of Genetics, Cell Biology, and Development, University of Minnesota, Minneapolis, Minnesota, United States of America; University of Texas-Houston Medical School, United States of America

## Abstract

Chromatin function depends on adequate histone stoichiometry. Alterations in histone dosage affect transcription and chromosome segregation, leading to growth defects and aneuploidies. In the fungal pathogen *Candida albicans*, aneuploidy formation is associated with antifungal resistance and pathogenesis. Histone modifying enzymes and chromatin remodeling proteins are also required for pathogenesis. However, little is known about the mechanisms that generate aneuploidies or about the epigenetic mechanisms that shape the response of *C. albicans* to the host environment. Here, we determined the impact of histone H4 deficit in the growth and colony morphology of *C. albicans*. We found that *C. albicans* requires at least two of the four alleles that code for histone H4 (*HHF1* and *HHF22*) to grow normally. Strains with only one histone H4 allele show a severe growth defect and unstable colony morphology, and produce faster-growing, morphologically stable suppressors. Segmental or whole chromosomal trisomies that increased wild-type histone H4 copy number were the preferred mechanism of suppression. This is the first study of a core nucleosomal histone in *C. albicans*, and constitutes the prelude to future, more detailed research on the function of histone H4 in this important fungal pathogen.

## Introduction

*Candida albicans* is a major human fungal pathogen and is the fourth most common cause of nosocomial bloodstream infections [1]. *C. albicans* is a common commensal of the skin and mucosa, and often causes superficial, non-life threatening infections at these sites [2]. However, in immune-compromised individuals *C. albicans* can cause systemic infections, which have a mortality rate of >30% even in patients undergoing antifungal therapy [3]. The steady increase in the population of immune-compromised individuals due to modern medical practices such as chemotherapy and organ transplantation, as well as because of the AIDS epidemic continues to provide niches for the development of *C. albicans* systemic and mucosal infections.

*C. albicans* pathogenesis has been increasingly linked to alterations in chromosome structure and dynamics. *C. albicans* strains with altered karyotypes are frequently isolated from clinical samples, from passage through mammalian hosts, and by growth in specific carbon sources or antifungals *in vitro* [4], [5]. *C. albicans* has a high tolerance to aneuploidies, perhaps because they provide a source for phenotypic variation, critical for survival and pathogenesis [6]. Aneuploidies are associated with antifungal resistance, metabolic changes, and mating [7]–[13]. Altered karyotypes have also been associated with variations in colony morphology [4], [6], [14], [15]. However, the mechanisms that promote ploidy changes and genomic rearrangements are not well understood.

Histone modifying enzymes and chromatin remodeling proteins also contribute to the regulation of pathogenesis traits. For example, mutants in histone deacetylases, methylases, acetyltransferases, and members of chromatin remodeling complexes show defects in yeast-hyphal transitions, white-opaque switching, adhesion to epithelial cells, and/or antifungal and stress resistance [16]–[23]. Therefore, changes in the structure and function of the chromatin leads to epigenetic defects and potentially to karyotypic variations that have a direct impact on *C. albicans* virulence.

Chromatin is a dynamic structure composed of DNA and DNA binding proteins that allows for an efficient storage and usage of the genetic information. The basic unit of chromatin architecture is the nucleosome, which is composed of the evolutionary conserved histones H2A, H2B, H3 and H4 assembled in a hetero-octamer of two H2A/H2B dimers and one H3/H4 tetramer. The DNA is wrapped around the nucleosome, constituting the first level of chromatin compaction. Due to this intimate relationship with the DNA, histones are involved in all processes associated with chromatin structure and function, including transcription, replication, DNA repair, recombination, and chromosome segregation. Histones participate in the regulation of these processes by providing a platform to transmit information to other proteins (e.g. DNA and RNA polymerases) through posttranslational modifications in their residues [24], [25] and through nucleosomal occupancy of regulatory regions in the DNA [26], [27]. Thus, histones constitute the primary regulators of chromatin activity.

Alterations in histone availability have profound effects on the cell. Unbalanced histone dimer stoichiometry causes defects in the segregation of mitotic chromosomes, increases recombination and genetic instability, and leads to sporulation defects in *Saccharomyces cerevisiae* [28]–[37]. Furthermore, incomplete nucleosomal occupancy due to histone dosage defects directly impacts transcriptional regulation [38]–[44]. Therefore, alterations in histone stoichiometry have pleiotropic effects in cells.

In this study we performed serial deletions of *C. albicans* histone H4 genes and determined the effect of a deficit in histone H4 on growth. We found that reduced histone H4 dosage caused a severe growth defect and the formation of colony morphology variants. *C. albicans* primarily counterbalanced the low dosage of histone H4 by increasing histone H4 gene copy number through the formation of aneuploidies. Suppression of the growth defect associated with low histone H4 dosage also restored colony morphology to the wild-type morphology. This is the first study on core histones in *C. albicans*, which provides background genetic information for future experiments that address the role of chromatin structure and function in *C. albicans* biology and pathogenesis.

## Materials and Methods

### Strains and plasmids

**Figure 1:**
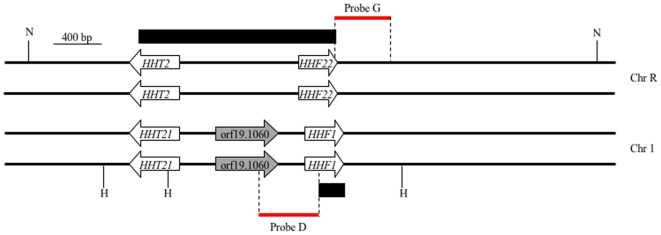
Genomic organization of the histone H3 and H4 loci in *Candida albicans*. The black boxes indicate the extent of the deleted regions in the mutants, which are replaced by the auxotrophic markers *ARG4*, *URA3*, *URA3-dpl200* or the *dpl200* loop-out. *HHF1* is deleted from nucleotide +114 to the STOP codon (65% of the gene) and the *HHF22-HHT2* cluster is deleted from the STOP codon of *HHF22* to nucleotide +329 of *HHT2* (80% of the *HHT2* gene). orf19.1060 is a possible spurious ORF. In red, the regions recognized by the probes D and G used for determining *HHF1* or *HHF22* copy dosage by Southern Blot are shown. H: HindIII; N: NcoI.

All strains used in this study are listed in Table 1. Strain DAY1069 was generated as follows. BWP17 was transformed with a *hht2-hhf22::URA3-dpl200* disruption cassette amplified in a PCR using primers HHT2-HHF22 5DR and HHT2-HHF22 3DR new (Table 2) to give strain DAY1067. DAY1067 was then transformed with a *hht2-hhf22::ARG4* disruption cassette amplified as above. The *hht2-hhf22* disruption cassettes delete the region from nucleotide +329 of *HHT2* to the stop codon of *HHF22* (Figure 1). The *URA3-dpl200* marker from strain DAY1069 was recycled by plating the cells in synthetic medium supplemented with 5-fluoroorotic acid (5-FOA) to obtain strain DAY1071. Strain DAY1072 was generated by replacing nucleotide +114 to the stop codon of one *HHF1* alleles in DAY1071 using the *hhf1::URA3* disruption cassette, which was amplified in a PCR using primers HHF1 5DR 100 in and HHF1 3DR new (Figure 1 and Table 2).

**Table 1:** Strains used in this study.

Strain	Parent/Background	Genotype	Reference
DAY1	BWP17	*ura3::λimm434/ura3::λimm434 his1::hisG/his1::hisG arg4::hisG/arg4::hisG*	[97]
DAY286	DAY1	*ura3::λimm434/ura3::λimm434 his1::hisG/his1::hisG ARG4::URA3::arg4::hisG/arg4::hisG*	[98]
DAY963	SC5314	Prototrophic clinical isolate	[99]
DAY1066	DAY1	*ura3::λimm434/ura3::λimm434 his1::hisG/his1::hisG arg4::hisG/arg4::hisG HHF1/hhf1::URA3-dpl200*	This study
DAY1067	DAY1	*ura3::λimm434/ura3::λimm434 his1::hisG/his1::hisG arg4::hisG/arg4::hisG HHT2-HHF22/hht2-hhf22::URA3-dpl200*	This study
DAY1068	DAY1066	*ura3::λimm434/ura3::λimm434 his1::hisG/his1::hisG arg4::hisG/arg4::hisG HHF1/hhf1::URA3-dpl200 HHT2-HHF22/hht2-hhf22::ARG4*	This study
DAY1069	DAY1067	*ura3::λimm434/ura3::λimm434 his1::hisG/his1::hisG arg4::hisG/arg4::hisG hht2-hhf22::ARG4/hht2-hhf22::URA3-dpl200*	This study
DAY1070	DAY1068	*ura3::λimm434/ura3::λimm434 his1::hisG/his1::hisG arg4::hisG/arg4::hisG HHF1/hhf1::dpl200 HHT2-HHF22/hht2-hhf22::ARG4*	This study
DAY1071	DAY1069	*ura3::λimm434/ura3::λimm434 his1::hisG/his1::hisG arg4::hisG/arg4::hisG hht2-hhf22::ARG4/hht2-hhf22::dpl200*	This study
DAY1072	DAY1071	*ura3::λimm434/ura3::λimm434 his1::hisG/his1::hisG arg4::hisG/arg4::hisG HHF1/hhf1::URA3 hht2-hhf22::ARG4/hht2-hhf22::dpl200*	This study
DAY1074	DAY1070	*ura3::λimm434/ura3::λimm434 his1::hisG/his1::hisG arg4::hisG/arg4::hisG hhf1::URA3/hhf1::dpl200 HHT2-HHF22/hht2-hhf22::ARG4*	This study
DAY1075	DAY1074	*ura3::λimm434/ura3::λimm434 his1::hisG/his1::hisG arg4::hisG/arg4::hisG hhf1::URA3/hhf1::dpl200 HHT2-HHF22/hht2-hhf22::ARG4*	This study
DAY1076	DAY1070	*ura3::λimm434/ura3::λimm434 his1::hisG/his1::hisG arg4::hisG/arg4::hisG hhf1::URA3/hhf1::dpl200 HHT2-HHF22/hht2-hhf22::ARG4*	This study
DAY1078	DAY1070	*ura3::λimm434/ura3::λimm434 his1::hisG/his1::hisG arg4::hisG/arg4::hisG hhf1::URA3/hhf1::dpl200 HHT2-HHF22/hht2-hhf22::ARG4*	This study
DAY1079	DAY1078	*ura3::λimm434/ura3::λimm434 his1::hisG/his1::hisG arg4::hisG/arg4::hisG hhf1::URA3/hhf1::dpl200 HHT2-HHF22/hht2-hhf22::ARG4*	This study
DAY414 (L40)	*S. cerevisiae*	*MATα his3Δ200 trp1-901 leu2-3, 112 ade2 LYS2::(lexAop)_4_-HIS3 URA3::(lexAop)_8_-lacZ GAL4*	[100]

**Table 2:** Primers used in this study.

Name	Sequence (5′to 3′)	Reference
HHF1 5 DR 100 in	5′-taaacgtcacagaaagattttaagagataacattcaaggtattacaaaaccagctatcagtttcccagtcacgacgtt	This study
HHF1 3 DR new	5′-ttaatactatacaataaagaaaacgaactaaaaagacaattagaaatacaacccagtttagtggaattgtgagcggata	This study
HHT2-HHF22 5 DR	5′-cttctagctaattgcatatctttcttttgaatggtaactctcttagcatggatagcacactttcccagtcacgacgtt	This study
HHT2-HHF22 3 DR new	5′-taatctaaaaatacagttatcatgaatcgaaaaacataaagaaaagaagatatttctttagtggaattgtgagcggata	This study
HHF1 5 detect	5′-tcttagtgtaaggaacctcc	This study
HHF1 3 detect	5′-acgattataaaggagaaggtg	This study
HHT2-HHF22 5 detect	5′-aaatgtccaataccagcacc	This study
HHT2-HHF22 3′detect-new	5′-ccgaaaataatttgcttgccttgcc	This study
HHF1 5′ fragm DDB78	5′-acgacggccagtgaattgtaatacgactcactatagggcggctcactcttagtgtaaggaacctcc	This study
HHF1 5′fragm 3′	5′-ctgatagctggttttgtaatacc	This study
HHF1 3′fragm 5′	5′-actgggttgtatttctaattgtc	This study
HHF1 3′ fragm DDB78	5′-aagctcggaattaaccctcactaaagggaacaaaagctggtctcagtgagctgttacgaggc	This study
HHF1 5′fragm 5	5′-ctcactcttagtgtaaggaacctcc	This study
HHF1 5′ 100 in for DDB78	5′-taaacgtcacagaaagattttaagagataacattcaaggtattacaaaaccagctatcaggggcgaattggggagctccc	This study
HHF1 3′ for DDB78	5′ -ttaatactatacaataaagaaaacgaactaaaaagacaattagaaatacaacccagtttacgataagcttcatctagaagg	This study
HHF22 5 SB	5′-accttgtatggtttcggtgg	This study
HHF22 3 detect	5′-gttattcggttagaaagcgg	This study

DAY1068 was generated by transforming strain BWP17 with the *hhf1::URA3-dpl200* disruption cassette (Figure 1) to generate strain DAY1066, followed by transformation with the *hht2-hhf22::ARG4* disruption cassette (Table 2). Strain DAY1070 was obtained by plating DAY1068 in synthetic medium supplemented with 5-FOA to recycle the *URA3-dpl200* marker. Strains DAY1074, DAY1076 and DAY1078 were generated by partially deleting the last *HHF1* copy from DAY1070 using an *hhf1::URA3* disruption cassette amplified from plasmid DDB383 with primers HHF1 5′ fragm DDB78 and HHF1 3′ fragm DDB78. Plasmid DDB383 contains the disruption cassette bordered by additional *HHF1* flanking regions in order to increase the efficiency of integration into the *HHF1* locus. Strains DAY1075 and DAY1079 are large colony revertants of strains DAY1074 and 1078, respectively. The genotypes of the strains and the correct integration of the disruption cassettes were verified by the PCR using primers HHF1 5 detect, HHF1 3 detect, HHT2-HHF22 5 detect and HHT2-HHF22 3′detect-new that flank the integration site (Table 2), and by Southern blot.

Plasmid DDB383 was generated by *in vivo* recombination as follows. An *hhf1::URA3* disruption cassette was amplified in a PCR from DDB245 [45] using primers HHF1 5′ 100 in for DDB78 and HHF1 3′ for DDB78 (Table 2). The two flanking *HHF1* regions of 570 bp and 523 bp with homology upstream (including the first 113 nucleotides of *HHF1*) and downstream of *HHF1*, respectively, were amplified in two high fidelity PCRs (Pfu Turbo DNA polymerase, Stratagene) from BWP17 genomic DNA using the primer pairs HHF1 5′ fragm DDB78 and HHF1 5′ fragm 3′, and HHF1 3′ fragm DDB78 and HHF1 3′ fragm 5′, respectively. The three PCR products were co-transformed with a NotI/EcoRI double digestion of DDB78 into the Trp^-^ *Saccharomyces cerevisiae* L40 strain to generate DDB383.

### Media and growth conditions

*C. albicans* was routinely grown at 30°C in YPD supplemented with uridine (2% bacto-peptone, 1% yeast extract, 2% dextrose, and 80 µg ml^−1^ of uridine). Mutants were selected on synthetic medium (0.17% yeast nitrogen base without ammonium sulfate (Q-BioGene), 0.5% ammonium sulfate, 2% dextrose, and supplemented with a dropout mix containing amino and nucleic acids except those necessary for the selection [46]. Solid media were prepared by addition of 2% Bacto-agar.

### Southern blot and comparative genomic hybridizations

For the *HHF1* and *HHF22* dosage experiments, genomic DNA was digested with HindIII or NcoI, respectively (Figure 1), separated in a 1.2% agarose gel by electrophoresis, and transferred by capillary action to a nylon membrane. Probes D (for *HHF1*) and G (for *HHF22*) were PCR amplified from *C. albicans* BWP17 genomic DNA using primer pairs HHF1 5′ fragm 5′ and HHF1 5′ fragm 3′, and HHF22 5′ SB and HHF22 3′ detect, respectively (Figure 1 and Table 2). The probes were radiolabelled with [α-^32^P]-dCTP using the Prime-a-Gene labeling system (Promega). Blots were developed with a phosphoimager (STORM system). Densitometry analysis of the images was performed using ImageJ 1.30v (Wayne Rasband, NIH, USA). For comparative genomic hybridizations (CGH), genomic DNA was prepared from *C. albicans* strains grown overnight to saturation in 5 ml YPAD medium using phenol/chloroform as described [47]. 3 µg of DNA was digested with HaeIII (Invitrogen), labeled with Cy3 (experimental strains) or Cy5 (reference strain, SC5314), and hybridized to microarrays as described previously [48]. The microarrays were printed in-house and contain 14,688 total spots, representing 6175 ORFs, designed using Assembly six *C. albicans* ORFs [49] and updated with Assembly 19 ORFs. Arrays were scanned (ScanArray 5000) using QuantArray v.2.01 software (GSI Lumonics, Watertown, MA). Data were analyzed using GenePixPro 5.1 and GeneTraffic 3.1. The average mean log2 ratio average of two duplicate spots per microarray slide was calculated and plotted as a function of chromosome position using Chromosome_Map [48].

## Results and Discussion

### Histone H3 and H4 genes in *Candida albicans*

Several characteristics of histones H3 and H4 prompted us to focus on them for the purpose of this study. First, histones H3 and H4 show greater conservation than histones H2A and H2B [50]. Second, the H3/H4 tetramer has a more critical role in nucleosome assembly and chromatin condensation, as the H3/H4 tetramer interacts more strongly with the central portion of the nucleosomal DNA than the H2A/H2B dimers [51], [52], and it has the ability to block transcriptional elongation *in vitro* [53]. Third, the effect of histone H3 and H4 posttranslational modification is better understood and includes modifications in both the amino terminal tail and globular core domains [54], [55]. Based on the higher conservation, the prominent role in chromatin structure, and the extensive knowledge on histone H3 and H4 posttranslational modifications, we focused on the histone H3-H4 cluster and, in particular, on histone H4, for reasons discussed below.

**Figure 2:**
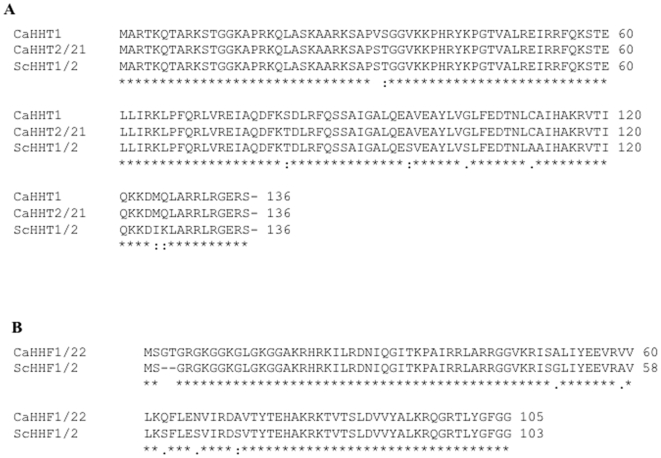
Amino acid sequence alignment of histone H3 and H4 genes. Comparison of the amino acid sequences of histone H3 (A) and histone H4 (B) alleles from *C. albicans* and *S. cerevisiae* using ClustalW.

Most eukaryotes, including *C. albicans*, contain multiple copies of histone H3 and H4 genes, which are generally organized in clusters [56]. The *S. cerevisiae* genome contains two genes, *HHT1* and *HHT2*, which encode identical proteins for the canonical histone H3 isoform. The *C. albicans* genome contains three genes that encode histone H3: *HHT1* (orf19.6791), *HHT2* (orf19.1853), and *HHT21* (orf19.1061). *HHT2* and *HHT21* encode identical histone H3 isoforms, whereas *HHT1* encodes a histone H3 with three differences when compared to *HHT2/21*: a non-conserved S32V change, and two conserved S33T and S81T changes (Figure 2A). When compared to *S. cerevisiae HHT1/2*, there are five amino acid differences, which are found in all three *C. albicans* histone H3 genes and are located in the C terminal half of the protein (Figure 2A).

Similar to *S. cerevisiae* and *Schizosaccharomyces pombe* [57], [58], *C. albicans HHT2* and *HHT21* are divergently transcribed from the histone H4 genes *HHF22* (orf19.1059) and *HHF1* (orf19.1854), respectively. *HHT2-HHF22* is on chromosome R; *HHT21-HHF1* is on chromosome 1 (Figure 1). *HHT1*, on chromosome 3, is unique in that it is not paired with a histone H4 gene. Unpaired histone genes are also observed in other fungi, including *S. pombe*, which has an unpaired histone H2A [57], [59]. *HHT2* and *HHT21* are located 900–1000 bp from their cognate histone H4 gene and likely share the same promoter. However, we noted a potential open reading frame (ORF), orf19.1060, within the *HHT21-HHF1* intergenic region (Figure 1). This ORF can encode a protein of 169 residues. However, this predicted protein has no significant homology to other proteins or identifiable motifs, suggesting that orf19.1060 is a spurious ORF. Therefore, the genomic arrangement of the H3/H4 cluster in *C. albicans* is similar to *S. cerevisiae*, except for the presence in *C. albicans* of a single unpaired, divergent third histone H3 gene.

Since Hht1 has a divergent amino acid sequence compared to Hht2/21 and is expressed independent of a histone H4, we propose that Hht1 is a histone H3 variant. Histone variants are usually replication-independent, diverge from the canonical histone sequences, and have a different function [60], [61]. For example, *Drosophila melanogaster* and vertebrates express histone H3.3, a canonical histone H3 variant that differs in four amino acids, three of which are clustered in the histone fold domain [62]–[65]. Although all three *C. albicans* histone H3s are of the H3.3 class and the amino acid differences in Hht1 are not within the histone fold domain, Hht1 might still constitute a histone H3 variant. In particular, the S32V change in Hht1 could affect epigenetic regulatory events as it eliminates a potential phosphorylation site in the N-terminus tail of Hht1 [66], [67]. Thus, *HHT1* may encode a histone H3 homomorphic variant.

The *C. albicans* genome contains two genes, *HHF1* and *HHF22*, that encode identical histone H4 proteins. The *S. cerevisiae* genome also encodes two identical histone H4 proteins. Comparison of *C. albicans* Hhf1/22 to *S. cerevisiae* Hhf1/2, revealed seven amino acids differences (Figure 2B). Therefore, the genomic arrangement of histone H4 genes in *C. albicans* is similar to *S. cerevisiae*. Since *C. albicans* contains three non-allelic histone H3 genes as well as a heteromorphic histone H3 variant Cse4, which replaces histone H3 in centromeric chromatin [68], [69], we focused our studies on histone H4, which is encoded by two genes and is present in all nucleosomes.

### Reduced histone H4 dosage impairs *C. albicans* growth

Previous work in *S. cerevisiae, S. pombe*, and *D. melanogaster* showed that alterations in the dosage of the core nucleosomal histones can lead to pleiotropic phenotypes, including growth and cell cycle defects, chromosomal and telomere instability and gene expression deregulation [28], [29], [34], [36], [38], [39], [41], [70]–[72]. In order to understand the effects of altering histone H4 dosage in *C. albicans*, sequential deletions of three of the four histone H4 alleles were performed.

**Figure 3:**
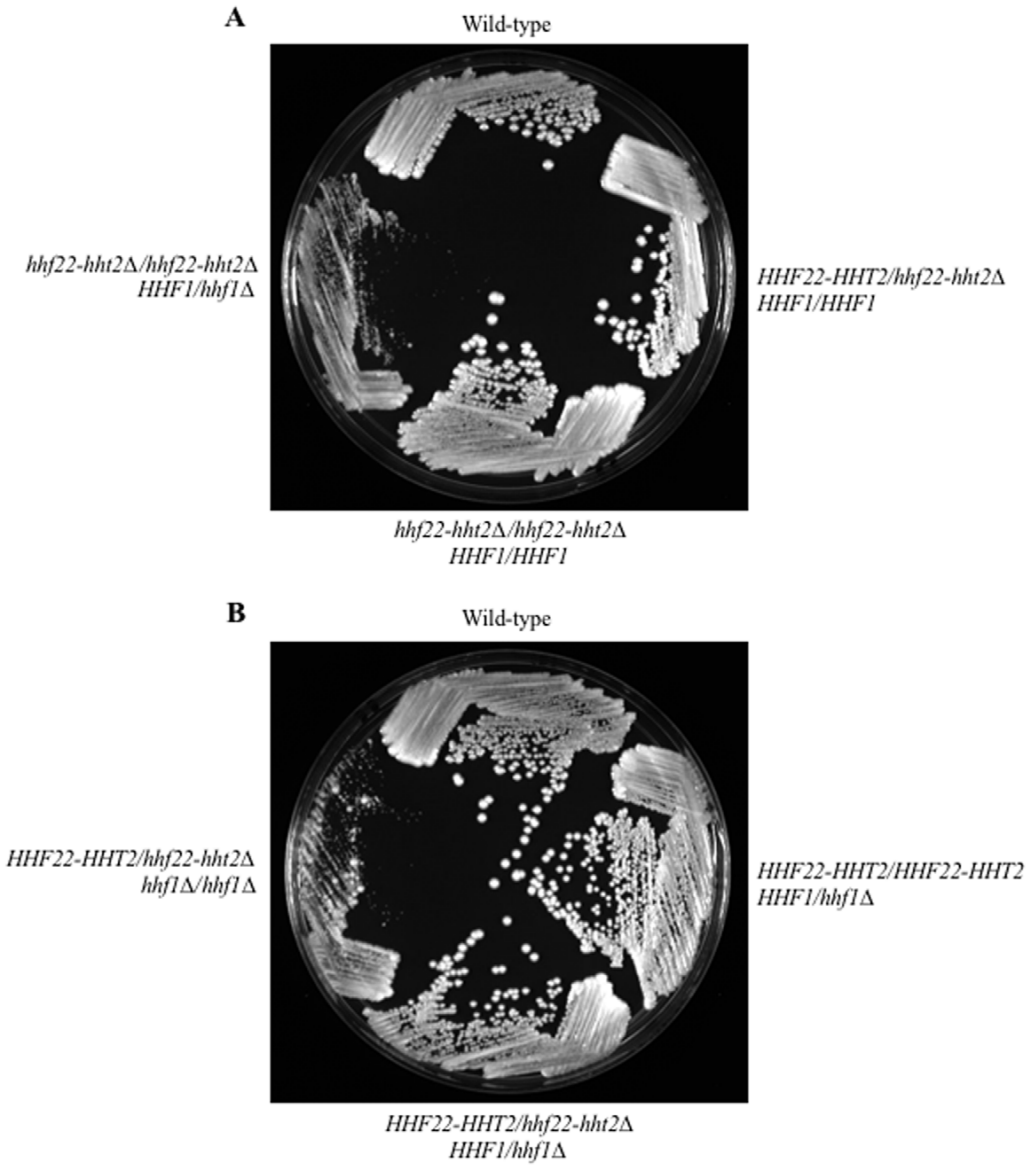
Growth of (A) wild-type (DAY286), *HHF22-HHT2/hhf22-hht2Δ* (DAY1067), *hhf22-hht2Δ/hhf22-hht2Δ* (DAY1069), and *hhf22-hht2Δ/hhf22-hht2Δ HHF1/hhf1Δ* (DAY1072) or (B) wild-type (DAY286), *HHF1/hhf1Δ* (DAY1066), *HHF1/hhf1Δ HHF22-HHT2/hhf22-hht2Δ* (DAY1068), and *hhf1Δ/hhf1Δ HHF22-HHT2/hhf22-hht2Δ* (DAY1074) histone H4 mutants in rich medium. All strains were grown overnight at 30°C in liquid YPD, streaked on YPD, and incubated at 30°C for 48 hrs.

First, we generated a deletion of the *HHF22-HHT2* region, which lacks additional ORFs (Figure 1). *HHF22* and *HHT2* were simultaneously deleted in order to reduce any potentially harmful effects of changing the histone H3/histone H4 ratio [36], [41], [71], [73]. The entire *HHF22* ORF, 80% of the *HHT2* ORF, and the intergenic region were replaced by an auxotrophic marker cassette (Figure 1). We were able to generate both *HHF22-HHT2/hhf22-hht2Δ* heterozygous and *hhf22-hht2Δ/Δ* homozygous strains, and these strains had no overt growth or morphological phenotypes (Figure 3A). This suggests that Hhf22 and Hht2 are not essential for growth.

*S. cerevisiae* diploids containing a single H3-H4 locus have a longer generation time and a more prolonged G1 phase than wild-type cells [70]. Further, deletion of *HHT2*-*HHF2* in *S. cerevisiae*, but not *HHT1*-*HHF1*, causes greater minichromosome loss compared to wild-type [70], indicating that, although both H3-H4 loci are generally functionally redundant, there are differences between both loci. To determine if *C. albicans* has a different requirement for *HHF1* or *HHF22* for growth, we constructed a strain in which both copies of *HHF1* were deleted. We were readily able to generate *HHF1/hhf1Δ* and *hhf1Δ/Δ* strains, and these strains did not show growth or morphological defects (Figure 3B and data not shown), indicating that Hhf1 is also not essential for growth in *C. albicans*. Therefore, unlike in *S. cerevisiae*, in *C. albicans* the presence of either one of the histone H4 gene is sufficient to ensure normal growth and colony morphology.

**Figure 4:**
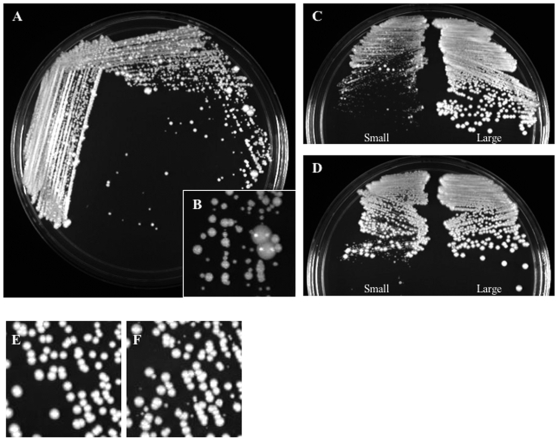
Growth defect and phenotypic instability of mutants containing a single allele of histone H4. A small colony of a histone H4 mutant (DAY1072) was re-streaked on YPD medium and incubated 4 days at 30°C (A), close-up picture (B). Re-isolated small (C) and large (C, E) colonies of *hhf22-hht2Δ/hhf22-hht2Δ HHF1/hhf1Δ* (DAY1072), and small (D) and large (D, F) colonies of *HHF22-HHT2/hhf22-hht2Δ hhf1Δ/hhf1Δ* (DAY1074 and DAY1079) incubated on YPD for 48 hrs at 30°C.

When we attempted to mutate one copy of *HHF1* in the *hhf22-hht2Δ/Δ* background, we only recovered a single homologous recombinant (1 homologous recombinant/395 transformants screened). Similarly, when we attempted to mutate the remaining copy of *HHF22-HHT2* from a *HHF22-HHT2/hhf22-hht2Δ HHF1/hhf1Δ* double heterozygote, we only recovered homologous recombinants 7% of the time (32 homologous recombinants/437 transformants screened). However, all of these recombinants retained a wild-type *HHF22-HHT2* copy and arose by a marker exchange (29/32) or a by a increased *HHF22-HHT2* copy number (3/32). To increase the rate of homologous recombination, we generated an *hhf1::URA3* disruption cassette containing regions of homology ∼9x larger than the original cassettes (Table 2). Using this extended disruption cassette we increased homologous recombination in the *hhf22-hht2Δ/Δ* strain to 50% (11/22). All *hhf22-hht2Δ/Δ HHF1/hhf1Δ* transformants had a pronounced growth defect (Figure 3A) and gave rise to both smooth and wrinkly colonies of heterogeneous size (Figure 4A and B). The difficulty in eliminating a third histone H4 gene and the severe growth defects observed in the mutant with only one *HHF1* copy suggested that *C. albicans* might require at least two copies of histone H4 for normal growth. Alternatively, *HHF22* might be the primary histone H4 gene in *C. albicans*.

**Figure 5:**
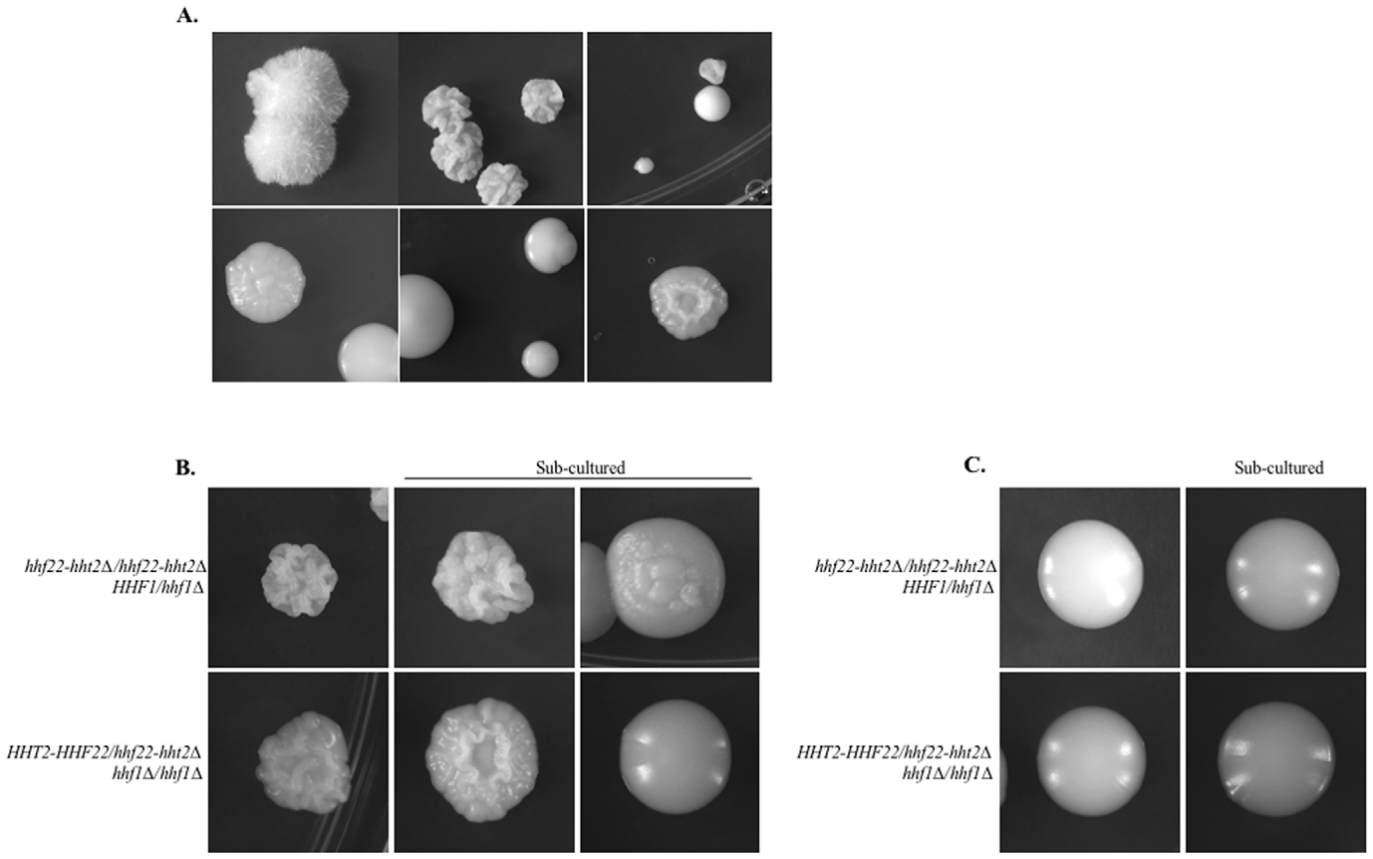
Histone deficit causes alterations in colony morphology. Examples of morphologically altered colonies that arise in mutants with only one *HHF1* (DAY1072) or one *HHF22* (DAY1079) allele after overnight incubation at 30°C on YPD (A). Partial penetrance of the morphological change in the small colonies (B). Stable morphology of the large smooth suppressor colonies after subculturing (C).

If *HHF22* is indeed the primary histone H4 gene in *C. albicans*, we predicted that we could construct an *HHF22/hhf22Δ hhf1Δ/Δ* mutant without impacting growth. To construct this mutant, we eliminated the last *HHF1* copy from a *HHF22-HHT2/hhf22-hht2Δ HHF1/hhf1Δ* double heterozygous strain. As before, the use of longer regions of homology increased the rate of homologous recombinants (44/45). However, in most cases (40/44) the wild-type *HHF1* copy was retained. While the *HHF22-HHT2/hhf22-hht2Δ HHF1/hhf1Δ* strain had no overt growth defects compared to wild-type colonies, the four *HHF22-HHT2/hhf22-hht2Δ hhf1Δ/Δ* mutants obtained had severe growth defects (Figure 3B). *HHF22-HHT2/hhf22-hht2Δ hhf1Δ/Δ* strains also gave rise to heterogeneous colony sizes with both smooth and wrinkly morphologies (Figure 4 and 5). The similar growth defects in the *HHF22-HHT2/hhf22-hht2Δ hhf1Δ/Δ* and *hhf22-hht2Δ/Δ HHF1/hhf1Δ* strains suggest that *HHF22* is not the primary histone H4 gene. Rather, *C. albicans* growth is dependent on the presence of at least two copies of histone H4.

We noticed that both *hhf22-hht2Δ/Δ HHF1/hhf1Δ* and *HHF22-HHT2/hhf22-hht2Δ hhf1Δ/Δ* strains gave rise to larger colonies at a low frequency (Figure 3 and 4). We considered the possibility that the larger colonies contained stable suppressor mutations. To address this, we re-isolated small and large colonies on fresh medium. When re-isolated, *hhf22-hht2Δ/Δ HHF1/hhf1Δ* small colonies gave rise to primarily small colonies with distinct morphologies (Figure 5), and a few large and always smooth colonies (Figure 4C). However, *hhf22-hht2Δ/Δ HHF1/hhf1Δ* large colonies gave rise to uniformly large and smooth colonies (Figure 4C). Re-isolation of small colonies from the *HHF22-HHT2/hf22-hht2Δ hhf1/hhf1Δ* strain also gave rise to small colonies with distinct morphologies, and to large colonies (Figure 4D and 5). In contrast to the *hhf22-hht2Δ/Δ HHF1/hhf1Δ* strain (Figure 4E), re-isolation of large colonies from the *HHF22-HHT2/hhf22-hht2Δ hhf1Δ/Δ* strain gave rise to primarily large colonies, but also occasionally to small colonies (Figure 4F). When re-isolated, these small colonies behaved like the parental *HHF22-HHT2/hhf22-hht2Δ hhf1Δ/Δ* small colonies (data not shown), indicating that the suppressor phenotype may be reversible. These results suggest that the severe growth defect caused by the low dosage of both *HHF1* and *HHF22* confers a strong selective pressure for the generation of secondary suppressor mutations, which restore growth. Further, the differences observed in the emergence of small colonies during re-isolation of large colonies in both type of mutants is likely a consequence of the stability of the suppressors that arose in the mutants.

### Aneuploidy as a mechanism for H4 dosage compensation in *C. albicans*

Two copies of histone H4 are necessary and sufficient for wild-type growth. Thus, we reasoned that the increased colony size and phenotypic stability observed in the large suppressor colonies reflected an increase in histone H4 copy number. Since aneuploidies are common in *C. albicans* strains [5], [7], [8], [10], [11], [13], [48], [74]–[78], we hypothesized that the large suppressor colonies had duplicated the remaining histone H4 gene, thereby restoring the number of histone H4 alleles to two, which supports normal and phenotypically stable growth (Figure 3). The duplication of large DNA fragments, including whole chromosomes, as a mechanism to suppress a slow growth defect has also been described in *S. cerevisiae* [79].

**Figure 6:**
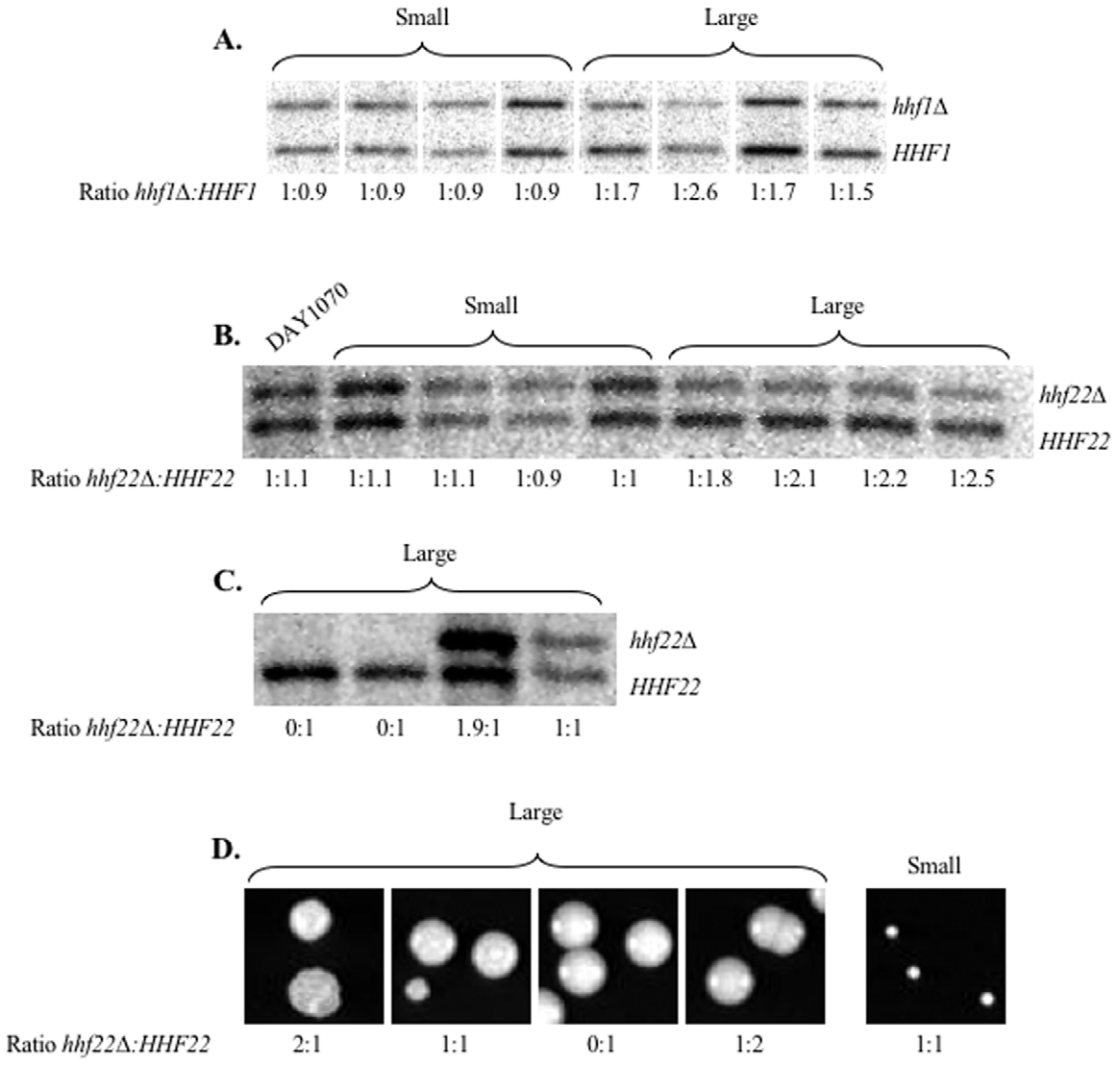
Quantitative Southern blot of histone H4 alleles. DNA samples were obtained from 30°C overnight cultures of small and large colonies isolated from (A) *hhf22-hht2Δ/Δ HHF1/hhf1Δ* (DAY1072) and (B and C) *HHF22-HHT2/hhf22-hht2Δ hhf1Δ/Δ* (DAY1074, DAY1075, DAY1076, DAY1078 and DAY1079). Due to the formation of suppressors, the proportion of small to large colonies was verified for each overnight culture by colony count on YPD to ensure that the DNA extraction was representative of a small or a large colony population. Overnight cultures from small colonies with a minimum of 80% of small colonies were used to prepare DNA for the Southern blot. Representative colonies from these plates corresponding to different ratios of mutated to wild-type histone H4 ratio are shown (D). Probes D and G were used to detect the alleles: *hhf1::URA3* and *HHF1*, and *hhf22::ARG4* and *HHF22*, respectively. *HHF22-HHT2/hhf22-hht2Δ HHF1/hhf1Δ* (DAY1070) was used in (B) as a control for a 1:1 mutated to wild-type *HHF22* ratio.

In order to determine if the large colonies of the *hhf22-hht2Δ/Δ HHF1/hhf1Δ* and *HHF22-HHT2/hhf22-hht2Δ hhf1Δ/Δ* mutants had increased the number of wild-type H4 allele copies, we performed quantitative Southern blot analysis. Two probes, Probe D and Probe G, were designed to anneal equally to both the wild-type and the mutated versions of *HHF1* or *HHF22*, respectively (Figure 1). Densitometry analysis was performed on Southern blots of small and large colonies isolated from the mutants to determine the ratio of mutated to wild-type histone H4 allele (Figure 6). We included the *HHF22-HHT2/hhf22-hht2Δ HHF1/hhf1Δ* heterozygous strain DAY1070 as a control, which had a 1∶1.1 *hhf22Δ/HHF22* ratio by quantitative Southern blot (Figure 6B). Indeed, the small colonies of the histone mutants retained a 1∶0.9–1.1 ratio of mutated to wild-type H4 alleles while the large colonies presented a 1∶1.5–2.6 ratio of mutated to wild-type H4 alleles (Figure 6A and 6B). Thus, the large colonies of the *hhf22-hht2Δ/Δ HHF1/hhf1Δ* and *HHF22-HHT2/hhf22-hht2Δ hhf1Δ/Δ* mutants analyzed showed an increase in DNA associated with the wild-type histone H4 compared to the small colonies. Thus, the suppressor colonies arise by increasing the genomic dosage of histone H4.

**Figure 7:**
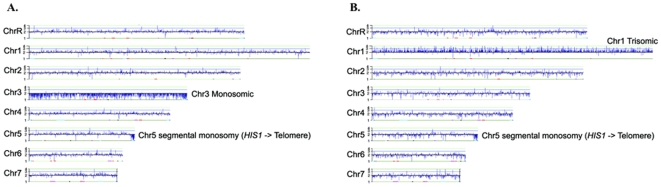
CGH analysis of a *hhf22-hht2Δ/hhf22-hht2Δ HHF1/hhf1Δ* (DAY1072) small colony (A) and a spontaneous large colony suppressor (B). Genomic DNA was purified from both a DAY1072 small colony and large colony and compared to genomic DNA from SC5314 (DAY963). The DAY1072 small colony is monosomic for Chromosome 3 while the DAY1072 large colony is trisomic for Chromosome 1. Both isolates have a short segmental aneuploidy on Chromosome 5R, which is present in the strain background [12], [48].

We reasoned that an increase in histone H4 copy number could involve either a segmental aneuploidy or a trisomy. Using comparative genome hybridization (CGH) arrays, we found that, as expected, a small colony of *hhf22-hht2Δ/Δ HHF1/hhf1Δ* had a diploid content of chromosome 1 (Figure 7). However, CGH analysis of a large suppressor colony derived from the *hhf22-hht2Δ/Δ HHF1/hhf1Δ* small colony revealed a trisomy of chromosome 1. Thus, a reduction in histone H4 dosage causes a severe growth defect that can be overcome through whole chromosome aneuploidy to increase histone H4 copy number. While we cannot rule out segmental aneuploidy as a mechanism to restore histone H4 dosage, whole chromosome aneuploidy is clearly one mechanism that can restore histone H4 dosage.

We noted two additional aneuploidies in our CGH analysis. First, a segmental monosomy of one end of chromosome 5 in both the small and large colony (Figure 7). This is an attribute of the RM1000 background from which these strains are descended, which is known to have a stable deletion in one arm of chromosome 5 [12], [48]. Second, a whole chromosome monosomy in chromosome 3 was observed in the *hhf22-hht2Δ/Δ HHF1/hhf1Δ* small colony, but not the large suppressor colony derivative. Defects on histone dosage has been implicated in the generation of aneuploidies and chromosome instability, and this may reflect evidence of that phenomenon in *C. albicans*. However, it is also possible that reduced chromosome 3 dosage provides some advantage to cells containing one histone H4 locus.

It is noteworthy that not all large colonies gave the expected 1:∼2 ratio of mutant to wild-type histone H4 by quantitative Southern blot (Figure 6C). We found a 0∶1 ratio (arbitrarily set to 1 as there was no mutant allele to normalize to), which lost the mutant histone H4 allele, a 1∶1 ratio, which maintained the ratio of the starting strain, and a ∼2∶1 ratio, which duplicated the mutant histone H4 allele. The 0∶1 ratio found in large colonies indicates that the mutated version of the histone H4 was replaced with the wild-type histone H4 by mitotic recombination or by loss of the chromosome carrying the mutant allele followed by duplication of the remaining chromosome containing the wild type allele. The 1∶1 ratio found in large colonies indicates that either the strain became tetrasomic, duplicating both loci, or that there exist alternative suppressor mechanisms. The ∼2∶1 ratio found in large colonies indicates that the mutated version of the histone H4 was duplicated, perhaps causing a trisomy of part or all of chromosome R. This type of aneuploidy may restore growth, however we cannot rule out the possibility that chromosome R is found in a 4∶2 mutant to wild-type ratio in these cells. In fact this latter possibility seems to be supported by the increased *hhf22Δ* and *HHF22* signals observed in this sample (Figure 6C). We noted that the large colonies carrying a 0∶1, 1∶1, and 1∶2 ratio of mutant to wild-type histone H4 had the typical smooth morphology, but the colonies carrying a 2∶1 ratio showed a wrinkly top and were more heterogeneous in size (Figure 6D). These differences in colony morphology are in agreement with the formation of alternative karyotypes (see below). The 0∶1, 1∶1, and 2∶1 ratio for large colonies arose from the *HHF22-HHT2/hhf22-hht2Δ hhf1Δ/Δ* mutant but not from the *hhf22-hht2Δ/Δ HHF1/hhf1Δ* mutant, which suggests that Chromosome R is less stable than Chromosome 1.

Since maintenance of genomic integrity is critical for survival, cells have different mechanisms to compensate for histone dosage defects, including genomic rearrangements and, more commonly, transcriptional alterations. Genomic rearrangements have been observed in *S. cerevisiae*, which can increase histone H2A-H2B copy number by forming a small circular chromosome [80]. Dosage compensation through transcriptional up-regulation of histone gene expression has been observed both in *S. cerevisiae*, where the expression of one of the H2A-H2B loci (*HTA1-HTB1*) is regulated by the availability of histones H2A-H2B in the cell [73], as well as in *S. pombe* [81]. Thus, while we observed an increase in gene copy number in the large colonies screened, we cannot rule out the possibility that *C. albicans* can increase histone H4 availability by transcriptional mechanisms.

We observed that while the large colonies of the *hhf22-hht2Δ/Δ HHF1/hhf1Δ* strain were phenotypically stable, the large colonies isolated from the *HHF22-HHT2/hhf22-hht2Δ hhf1Δ/Δ* strain often gave rise to small colonies. This observation suggests that a Chromosome R trisomy (*HHF22*) is less stable than a Chromosome 1 trisomy (*HHF1*). Chromosome R has a variable electrophoretic mobility. These variations are found in natural isolates, in spontaneous morphological mutants, and in randomly selected colonies from a clonal population of a reference strain [82]–[84]. However, chromosome R variations are attributed to changes in the number of rDNA repeats, and not to ploidy changes [84], [85]. A random gain and subsequent loss of an extra copy of Chromosome R might explain the slightly unstable phenotype of the large colonies with only *HHF22*. Acquiring a third Chromosome R copy would provide a selective growth advantage because it increases histone H4 copy number. However, excess of rDNA synthesis, or other chromosome R associated loci, could be deleterious for cells [84], [86], [87]. Thus, some suppressor cells might randomly lose the extra Chromosome R copy and revert to a slow growth small colony phenotype. Trisomy in Chromosome 1 might not have as negative an effect on growth, explaining the more stable colony morphology of the suppressors from strains with only a single allele of *HHF1*. In fact, some stocks of the routinely used laboratory strain CAI-4 are stably trisomic for Chromosome 1 under non-stress conditions with no obvious effects on growth or colony morphology [74]. Therefore, the alterations in colony size observed in both histone H4 mutants could be attributed to the gain and loss of a segment or an entire copy of Chromosome R or Chromosome 1.

### Histone H4 deficit causes colony morphology changes

When growing on agar plates at 30°C, *C. albicans* normally forms smooth, round, cream-colored colonies composed of yeast cells. Wrinkly colonies are generally composed of a higher percentage of cells that are filamenting, a fact that explains why it is possible to exacerbate some colony morphology phenotypic differences at 37°C. Altered *C. albicans* colony morphologies have been detected in strains isolated from infected patients, from studies in mouse models, and can also be induced in the laboratory through UV irradiation and genetic manipulation [4], [14], [88]–[93]. The changes in colony morphology are a manifestation of underlying genomic changes that can involve a group of genes (like in the white-opaque switching) or major karyotypic rearrangements [9]. Thus, alterations in diverse factors can lead to changes in colony morphology.

When small, smooth colonies that carry only one H4 allele are sub-cultured onto a rich medium plate they give rise to colonies that have different morphologies (Figure 5A). The change in colony morphologies of small colonies was generally penetrant, although they also gave rise to colonies with other types of morphologies (Figure 5B). On the contrary, the large and smooth suppressor colonies did not produce colonies with altered morphology when they were sub-cultured, indicating that their colony morphology phenotype is stable (Figure 5C). One exception to this statement is the already mentioned formation of small colonies from re-streaking of the large colonies, which most likely arise by the loss of the duplicated chromosome R.

One explanation for the formation of semi-penetrant morphological variants in the histone mutants is karyotypic rearrangements. As previously mentioned, altered colony morphologies have been associated with altered karyotypes or with loss of heterozygozity [4], [9], [94]. Imbalances in histone dosage can lead to chromosome missegregation [28]–[31]. Further, the histone mutants grow slowly, a condition that might be more permissive to the accumulation and tolerance of aneuploidies [4]. This latter idea is supported by the presence of a monosomic chromosome 3 in a *hhf22-hht2Δ/Δ HHF1/hhf1Δ* small colony (Figure 7). Thus, karyotypic rearrangements favored by the combination of the slow growth and the nucleosomal deficit of the histone H4 mutants might be one mechanism behind the formation of colony variants.

*Candida albicans* is the most important human fungal pathogen, causing serious infections in immunosuppressed individuals. *C. albicans* has a diploid genome with an unexpectedly high level of heterozygozity, given the primarily clonal reproductive style of this organism [95]. The genome of *C. albicans* has a remarkably high tolerance for genomic rearrangements. The ability to thrive with an altered karyotype may provide a profound advantage to this organism, because it represents a potential source of genetic variation [5]. Karyotypic rearrangements and aneuploidies in *C. albicans* are associated with pathogenesis: they affect cellular and colonial morphology, increase metabolic diversity, are required for mating, and, importantly, constitute a mechanism of antifungal resistance. Histone modifying enzymes and chromatin remodeling proteins are also required for pathogenesis in *C. albicans* [16]–[22], [96]. The study of chromatin dynamics and structure in this fungus therefore is critical for understanding the nature of *C. albicans* pathogenicity and, furthermore, it may uncover potential targets for antifungal therapies. In this study, we have generated and characterized strains that can be used for future analysis of specific histone H4 mutant alleles, in order to begin to dissect the function and impact of epigenetic regulation in *C. albicans* lifestyle and pathogenesis.
